# Chromatin Accessibility Mapping Identifies Mediators of Basal Transcription and Retinoid-Induced Repression of *OTX2* in Medulloblastoma

**DOI:** 10.1371/journal.pone.0107156

**Published:** 2014-09-08

**Authors:** Matthew Wortham, Changcun Guo, Monica Zhang, Lingyun Song, Bum-Kyu Lee, Vishwanath R. Iyer, Terrence S. Furey, Gregory E. Crawford, Hai Yan, Yiping He

**Affiliations:** 1 Department of Pathology, The Pediatric Brain Tumor Foundation Institute, and The Preston Robert Tisch Brain Tumor Center at Duke, Duke University Medical Center, Durham, North Carolina, United States of America; 2 Duke Center for Genomic and Computational Biology, Duke University, Durham, North Carolina, United States of America; 3 Department of Molecular Biosciences, University of Texas at Austin, Austin, Texas, United States of America; 4 Department of Genetics, Department of Biology, Carolina Center for Genome Sciences, and Lineberger Comprehensive Cancer Center, The University of North Carolina at Chapel Hill, Chapel Hill, North Carolina, United States of America; 5 Department of Pediatrics, Division of Medical Genetics, Duke University Medical Center, Durham, North Carolina, United States of America; Albert Einsten College of Medicine, United States of America

## Abstract

Despite an emerging understanding of the genetic alterations giving rise to various tumors, the mechanisms whereby most oncogenes are overexpressed remain unclear. Here we have utilized an integrated approach of genomewide regulatory element mapping via DNase-seq followed by conventional reporter assays and transcription factor binding site discovery to characterize the transcriptional regulation of the medulloblastoma oncogene *Orthodenticle Homeobox 2* (*OTX2*). Through these studies we have revealed that *OTX2* is differentially regulated in medulloblastoma at the level of chromatin accessibility, which is in part mediated by DNA methylation. In cell lines exhibiting chromatin accessibility of *OTX2* regulatory regions, we found that autoregulation maintains OTX2 expression. Comparison of medulloblastoma regulatory elements with those of the developing brain reveals that these tumors engage a developmental regulatory program to drive *OTX2* transcription. Finally, we have identified a transcriptional regulatory element mediating retinoid-induced OTX2 repression in these tumors. This work characterizes for the first time the mechanisms of OTX2 overexpression in medulloblastoma. Furthermore, this study establishes proof of principle for applying ENCODE datasets towards the characterization of upstream *trans*-acting factors mediating expression of individual genes.

## Introduction

Medulloblastoma is the most common pediatric brain malignancy. The substantial comorbidities associated with the standard medulloblastoma treatment of resection, radiation, and chemotherapy necessitate the development of selective therapeutics. While Sonic Hedgehog (Shh) pathway inhibitors have shown promise in treating medulloblastomas exhibiting Shh pathway hyperactivity [1], these tumors comprise only a subset of medulloblastoma, and few consistent druggable targets have been described among Shh-independent variants of this tumor (e.g. Wnt subgroup, Group 3, and Group 4) [2]–[4]. Our previous studies have revealed the importance of the homeobox transcription factor OTX2 to the maintenance of non-Shh medulloblastomas [5]. OTX2 transcriptionally regulates cell cycle genes [6], [7] and the *MYC* oncogene [5], thereby contributing to medulloblastoma maintenance, and additionally can alter the dynamics of hindbrain progenitor cell migration and proliferation [8]. This gene is expressed ubiquitously in Shh-independent medulloblastomas [2], [5] and is required for viability by tumors of this subgroup. OTX2 thus represents an attractive potential drug target in this tumor type; however, as a ligand-independent transcription factor, inhibiting its function with standard small molecule approaches is challenging. With this in mind, we have sought to understand the transcriptional regulation of *OTX2* with the goal of identifying strategies of pharmacologically inhibiting its expression.

Otx2 (the mouse homolog of human OTX2) exhibits a dynamic expression pattern during embryonic development [9], [10], with its transcription being mediated by various well-characterized enhancers and *trans*-acting regulators [11]–[13]. Otx2 mRNA is expressed broadly at the epiblast stage and is progressively restricted to the anteriormost portion of the embryo as development proceeds [9]. The boundary of Otx2 expression sharpens at the presumptive border of the midbrain and hindbrain during neural tube regionalization, and as the brain fully matures, Otx2 expression is further limited to the retina, pineal gland, dorsal tectum, choroid plexus, and posterior cerebellum [9], [14]–[16]. With the exception of the choroid plexus and cerebellum, Otx2 expression seems to be inherited rather than induced *de novo* in most brain tissues as indicated by lineage tracing of Otx2-expressing cells [9].

Little is known about the mechanisms of OTX2 overexpression in medulloblastoma. Although a subset of medulloblastomas (∼21%) exhibit *OTX2* genomic copy number gain [5], nearly all non-Shh medulloblastomas (∼74% of all medulloblastomas) overexpress OTX2 [2], [5], [17]; thus, there remains a substantial proportion of these tumors that aberrantly express this oncogene in the context of normal *OTX2* genomic copy number. While OTX2 is expressed in some tissues giving rise to midbrain and hindbrain [9] as well as in a subset of cerebellar granule neuron precursors [8], [15], which are known cellular origins of medulloblastoma, it is unclear whether OTX2 expression is inherited from the tumor cell of origin or rather induced over the course of transformation. The transcriptional regulation of *OTX2* in medulloblastoma may thus reflect the regulatory landscape sustained from a particular developmental timepoint or rather an unrelated regulatory mechanism unique to this tumor type.

Taking a cue from its developmental regulation [18], we and others have demonstrated that retinoic acid can repress transcription of *OTX2* [19], [20] and inhibit medulloblastoma growth [19]–[21]. Clinical trials have been initiated to test the benefit of adjuvant retinoid therapy in these tumors. However, preclinical studies indicate that retinoids may lack efficacy in orthotopic xenografts [20], potentially due to compensatory pathways not active *in vitro* or to an inability to repress *OTX2* in this tissue context. Understanding the mechanism of OTX2 repression by retinoids could reveal strategies of reinforcing this therapeutic effect. To this end, we have also investigated transcriptional regulatory elements mediating retinoid-induced OTX2 repression in medulloblastoma cells.

The identification of transcriptional regulatory elements for individual genes is notoriously difficult [22]. While evolutionary conservation is frequently an indicator of some functional relevance, this parameter does not distinguish regulatory elements active at different developmental or physiological states, and many regulatory regions are not well-conserved [23], [24]. The assessment of DNA fragments driving reporter genes in transgenic animals, often using conservation as a guide, has proven critical for the mapping of developmental enhancers for various genes, including *OTX2* [11]–[13], [22], [25], [26]. However, this approach is not applicable to specific pathological entities, such as OTX2-expressing medulloblastoma, for which there are limited animal models amenable to transgenic reporter analysis.

In consideration of the experimental limitations to characterizing the transcriptional regulatory regions of *OTX2* in medulloblastoma, we have utilized an integrated approach based upon an emerging technology, DNase-seq, that permits genomewide mapping of active regulatory elements such as promoters, enhancers, repressors, insulators, and locus control regions [27]. First, we mapped DNase hypersensitive (DHS) sites comprising ∼400 kb surrounding the *OTX2* locus, which were then characterized for activity and specificity with reporter assays. Minimally-required regions of DHS sites of interest were then interrogated for the presence of transcription factor binding motifs to identify *trans*-acting factors regulating OTX2 expression, which were then experimentally validated. Finally, we investigated datasets characterizing the epigenome of various relevant central nervous system (CNS) tissues to draw parallels between OTX2 regulation in medulloblastoma and in the developing brain. In addition to characterizing OTX2 regulation in this particular context, this study also establishes a framework for hypothesis-driven experiments derived from ENCODE data as this endeavor continues to map chromatin modifications and other informative characteristics of transcriptional regulatory domains.

## Materials and Methods

### Tissue Samples

Brain tumor cell lines and frozen primary tumor samples were obtained from the Duke University Brain Tumor Center Tissue Bank.

### Cell culture, reporter assays, and gene expression experiments

Medulloblastoma and glioblastoma cells were obtained from Darell Bigner and were maintained as previously described [19]. For luciferase assays, 10^5^ cells were plated into each well of a 24 well plate, and cells were transfected with 1.3 µg of reporter plasmid and 67 ng of internal control plasmid pRL-CMV using Lipofectamine 2000, and 24 hours later cells were harvested and tested for luciferase reporter activity using the Promega Dual-Luciferase Reporter Assay System. All luciferase assays were carried out using transient transfection of circular plasmids. For siRNA experiments, cells were transfected with siRNA's at a final concentration of 100 nM. siRNA's were obtained from Ambion. siRNA sequences are as follows: Scramble: GAGUCAACCUUAUGAUACUtt, OTX2 #1 GGAGGUGGCACUGAAAAUCtt, OTX2 #2 GGACACUAAUUCAUCUGUAtt. OTX2-targeted siRNA's were previously validated to repress OTX2 protein expression using the same transfection conditions and cell lines [5]. Coordinates of DHS sites assessed for reporter activities are summarized in Table S1. mRNA levels were determined by qPCR using Kapa reagents and SYBR detection. Western blotting was carried out using a standard protocol and the following antibodies: R & D BAF1979 anti-OTX2 and Santa Cruz FL-335 anti-GAPDH. All-*trans* retinoic acid and cycloheximide was purchased from Sigma. 5-aza-2′deoxycytidine was purchased from MP Biomedicals.

### DNase treatment and analysis of hypersensitive sites

Medulloblastoma and glioblastoma cell nuclei were prepared with 1–5×10^7^ cells using NP40 concentrations optimized for each cell line. Nuclei were treated with increasing concentrations of DNase and then prepped for either DNase-seq or DNase-PCR as previously described [23], [27]. DNase-seq data was processed as previously described [23]. For DNase-PCR, 9 ng of genomic DNA was amplified by qPCR in a 20 uL reaction using Kapa reagents and the SYBR detection method. Standard curves were generated for each amplicon to quantify target concentration from linear Ct values. For each DNase concentration, target concentrations of experimental amplicons were compared to that of a DNase-resistant region to serve as a loading control and normalize for nonspecific DNase activity.

### Transcription factor motif search

The critical 13 bp fragment of DHS 4 was extended by 6 bp in either direction to yield a 25 bp fragment (TGTCTCCGGGATTAATTATGGGCAC) for subsequent motif discovery. TRANSFAC TFblast [28], JASPAR CORE [29], JASPAR HOMEO [29], and Consite [30] databases were queried using default settings. Protein-DNA interacting motifs were aligned manually to motifs identified in [31] using MapDraw. Google searches were performed with a 5 bp sliding window for every position of the 25 bp query fragment.

### Chromatin immunoprecipitation

Chromatin immunoprecipitation was performed using anti-OTX2 (BAF1979, R & D) or IgG control antibodies using the ChIP Assay Kit (Upstate Cell Signaling). Enriched DNA was quantified using qPCR as described above, and ΔCt was calculated relative to Line 1 primers.

### Bisulfite treatment

500 ng of DNA from each sample was bisulfite-treated using the EZ DNA Methylation Gold Kit (Zymo Research) following the manufacturer's protocol, and approximately 50 ng of bisulfite-treated or untreated DNA was amplified by PCR using Faststar Taq. Each CpG site was scored using DNAstar Seqman Software. Methylation-specific PCR was performed on 20 ng bisulfite-treated DNA using Platinum Taq. Primer sequences and genomic coordinates are listed in Table S2.

## Results

### DNase-seq identifies a DHS site pattern unique to medulloblastoma

**Figure 1:**
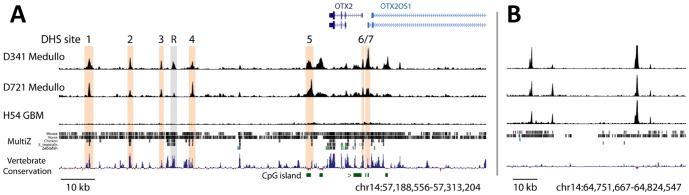
DNase landscape of OTX2-expressing and -nonexpressing cell lines. DNase hypersensitivity map of (A) the *OTX2* locus and (B) a region of chromosome 14 exhibiting DHS sites that are shared among the three cell lines shown. From top to bottom, tracks are as follows: Refseq genes, D341 medulloblastoma (Medullo) cells, D721 medulloblastoma cells, H54 glioblastoma (GBM) cells, MultiZ vertebrate alignments, PhyloP vertebrate conservation, CpG islands. Orange bars indicate regions of consistent and robust DNase hypersensitivity in the two medulloblastoma cell lines. Grey bar indicates DHS “R”, also found in retinoblastoma (Fig. S1A). Genomic coordinates are indicated below tracks. Carat indicates CpG island subjected to methylation analysis (Fig. 5 and Table S2). See Fig. S1A for extended viewing range.

In order to map the transcriptional regulatory elements of the *OTX2* gene, we performed DNase-seq on two medulloblastoma cell lines, D341 and D721, which overexpress OTX2 independent of genomic copy number gain [5], [19], [32], [33]. Both of these cell lines also express high levels of MYC (with D341 harboring genomic copy number gain and D721 exhibiting normal *MYC* copy number) [33], suggesting that these cell lines belong to the Group 3 medulloblastoma subtype [2]. Both cell lines exhibited a similar pattern of DHS site distribution flanking the *OTX2* locus, with many shared and prominent DHS sites present from 10 kb upstream to 80 kb downstream of the OTX2 long isoform transcriptional start site (Figs. 1A and S1A). All such DHS sites were highly conserved in mammals and most (with the exceptions of DHS sites 3 and 7) were alignable in vertebrates as divergent as Xenopus (Fig. 1 and Table S1). We noted that DNase hypersensitive regions were mostly absent outside of these clustered DHS sites (Fig. S1A) and that the canonical insulator protein, CTCF, bound strongly to regions flanking these clustered DHS sites (∼122 kb downstream and ∼50 kb upstream of *OTX2*, grey bars in Fig. S1B), indicating that *OTX2* regulatory regions are likely to reside within this range. Notably, many far distal regions were shared between medulloblastoma and other cell types (Fig. 1B) [34], as expected for this assay.

In contrast to medulloblastoma, the vast majority of cell lines derived from various normal and transformed tissues exhibited minimal to no DNase hypersensitivity in the vicinity of the *OTX2* gene (Fig. 1; refer to UCSC Genome Browser at http://genome.ucsc.edu for additional cell line data), in agreement with SAGE data indicating that OTX2 is expressed in a restricted subset of normal tissues and tumor types (e.g. medulloblastoma and retinoblastoma) [35]. Strikingly, many of the DHS sites in medulloblastoma were also present in a retinoblastoma cell line [Weri-Rb cells, data from [36]], with medulloblastoma exhibiting only two unique DHS sites (out of 10 prominent DHS sites) at the *OTX2* locus relative to retinoblastoma, and retinoblastoma exhibiting only four unique DHS sites (of 14 prominent DHS sites; Fig. S1A). Finally, a Ewing's sarcoma cell line (SK-N-MC, data from [36]) that expresses low but detectable levels of OTX2 exhibited one shared and three unique DHS sites at this locus relative to medulloblastoma (Fig. S1A). The correlated presence of clustered DHS sites in OTX2-expressing cell types is suggestive of previously-described clusters of regulatory elements (COREs) that cooperatively dictate cell type-specific gene expression patterns [23], [24]. DNase-seq analysis of ES cells, which appear to express OTX2 from the short isoform promoter (Fig. S1C) [10] revealed shared proximal DHS sites with medulloblastoma but lack of DNase hypersensitivity at medulloblastoma DHS sites 1–4 (Fig. S1A).

### Chromatin accessibility patterns are distinct between OTX2-expressing and -nonexpressing medulloblastomas

**Figure 2:**
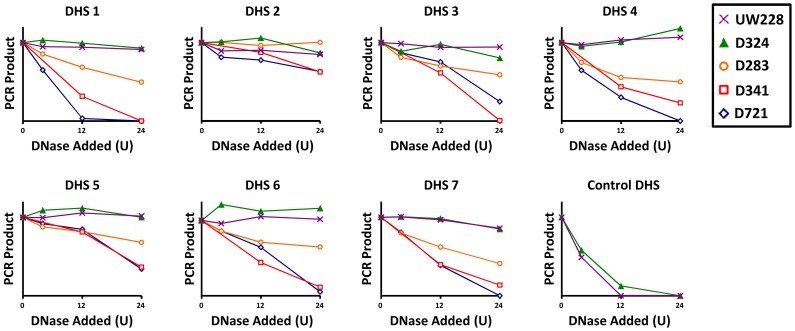
Validation of medulloblastoma DHS sites in a cohort of OTX2-expressing and -nonexpressing cells. Nuclei of medulloblastoma cell lines were treated with increasing concentrations of DNase (indicated as Units per reaction), and then the relative proportion of DNA remaining at the indicated regions were determined by qPCR. Open and solid markers indicate OTX2-expressing and -nonexpressing cell lines, respectively. See Fig. S2 for detailed graphs.

To determine the generalizability of this DHS site landscape among medulloblastomas, we implemented DNase-PCR (a targeted qPCR-based assessment of DNase sensitivity) to determine the DNase sensitivity of the seven most prominent DHS sites in a panel of medulloblastoma cell lines. To determine the specificity of DHS sites to OTX2-expressing cell lines, we included two cell lines that did not express OTX2 (UW228 and D324). With the exception of DHS 2, all DHS sites identified in the *OTX2* regulatory locus were DNase-hypersensitive in OTX2-expressing cells but DNase-resistant in OTX2-nonexpressing cell lines (Figs. 2 and S2). These findings indicate that among medulloblastomas, OTX2 expression is in part regulated by differential chromatin accessibility of regulatory regions surrounding the *OTX2* gene.

### DHS 4 uniquely exhibits enhancer activity specific to OTX2-expressing medulloblastomas

**Figure 3:**
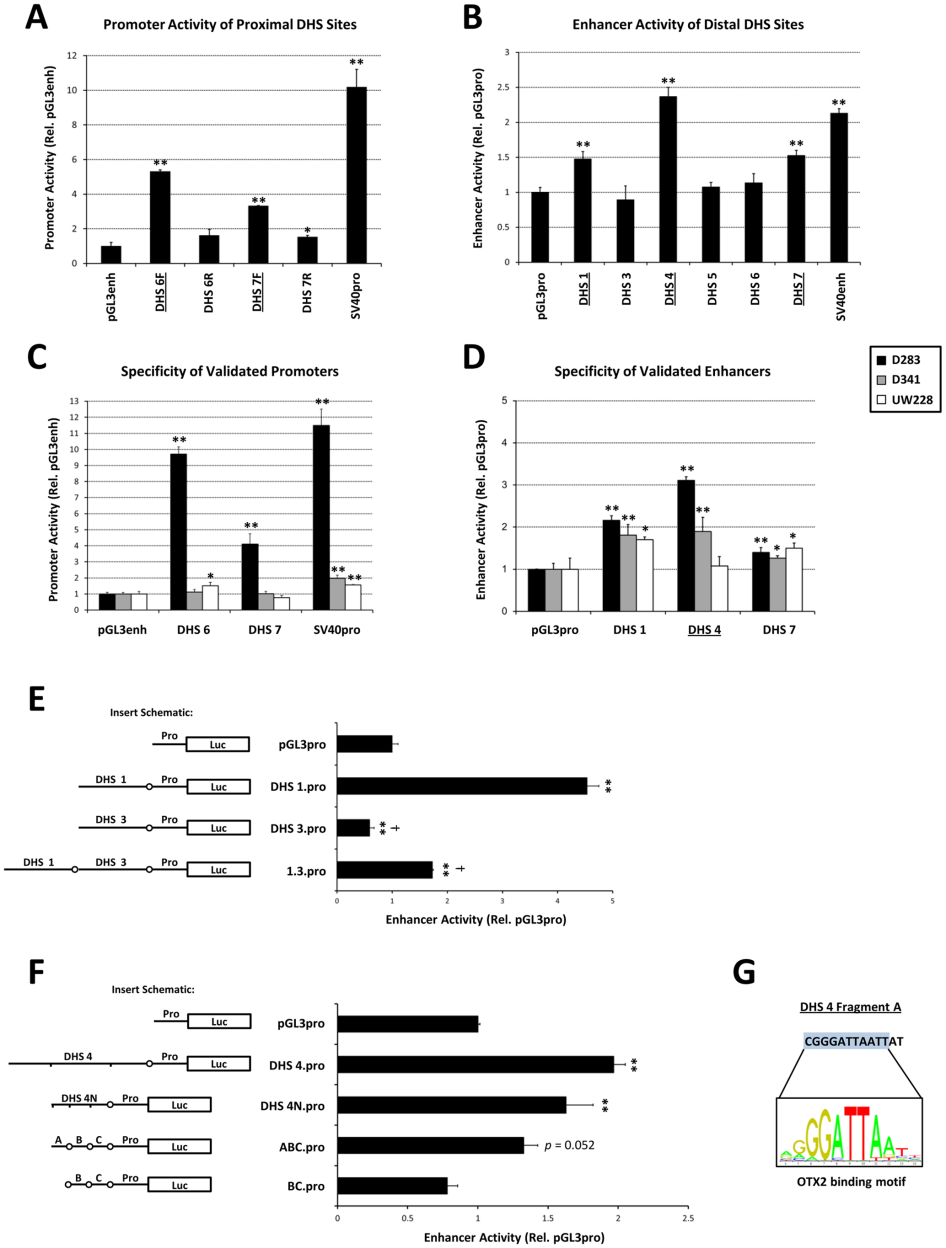
Functional assessment of DHS sites. Luciferase assays measuring (A) orientation-sensitive promoter activity of proximal (<2 kb from the TSS) DHS sites in D283 cells, (B) enhancer activity of all DHS sites in D283 cells, (C) promoter activity of functionally-validated proximal DHS sites [underlined in (A)] in OTX2-expressing (D283, D341) and -nonexpressing (UW228) medulloblastoma cells, (D) enhancer activity of functionally-validated DHS sites [underlined in (B)] in OTX2-expressing (D283, D341) and -nonexpressing (UW228) cells, (E) insulator activity of DHS 3 in OTX2-expressing medulloblastoma cells, and (F) enhancer activity of minimal fragments and deletion mutants of DHS 4 in OTX2-expressing medulloblastoma cells. (G) Alignment of the critical Fragment A region of DHS 4 with the OTX2 position weight matrix [28]. *p<0.05 relative to empty vector, **p<0.01 relative to empty vector, †p<0.05 relative to DHS 1.pro, ††p<0.01 relative to DHS 1.pro, Student's t-test. Error bars indicate standard deviation.

The activity of transcriptional regulatory elements can be controlled at the level of chromatin accessibility as well as through the activities of sequence-specific transcription factors. To measure the activities of such *trans*-acting factors in medulloblastoma cells, we assessed transcriptional regulatory function of the six validated DHS sites. ∼500 bp fragments encompassing each DHS site (see Table S1 for genomic coordinates) were cloned into luciferase reporter vectors and assessed for promoter activity (in the case of proximal DHS sites 6 and 7) or enhancer activity (all DHS sites) following transient transfection in OTX2-expressing D283 cells. These assays measure transcriptional activity from plasmid DNA and thus predominately reflect the activity of sequence-specific transcription factors. DHS sites 6 and 7 demonstrated strong, orientation-sensitive promoter activity in cooperation with a generic enhancer (Fig. 3A). Additionally, DHS sites 1, 4, and 7 exhibited enhancer activity when driving luciferase expression from a strong basal promoter (Fig. 3B). The specificity of these activities was then determined in OTX2-expressing (D283, D341) and -nonexpressing (UW228) cell lines (Fig. 3C–D). We identified variable promoter activities of DHS sites 6 and 7 among medulloblastoma cell lines (Fig. 3C), indicative of a role for tumor genetic background (independent of *OTX2* status) in affecting proximal regulatory element utilization in this tumor. However, testing activities of distal DHS sites revealed that the enhancer activity of DHS 4 closely associated with OTX2 expression status (Fig. 3D). These results suggest that a transcription factor regulating the activity of DHS 4 could influence OTX2 expression status in medulloblastoma.

Having observed that the insulator element CTCF bound to DHS 3 (Fig. S1B), we confirmed the ability of this fragment to partially block the enhancer activity of DHS 1 in a reporter assay (Fig. 3E), indicating that the enhancer activity of DHS 1 may be attenuated by DHS 3 in its native genomic context.

### OTX2 binds to and transactivates DHS 4

Considering that DHS 4 exhibited specific and consistent activity in OTX2-expressing medulloblastoma cells (Fig. 3C–D), we reasoned that the activity of *trans*-acting factors binding to this element may determine the transcriptional status of *OTX2* in medulloblastoma. To identify regulatory proteins mediating DHS 4 activity, we first sought to identify the critical sequence elements of this enhancer by generating progressive deletions within this fragment. This analysis revealed that Fragment A, a centrally-located 13 bp sequence, was critical for DHS 4 enhancer activity (Fig. 3F). Given the size of this sequence, it would be expected that scanning this fragment for transcription factor binding motifs would yield the *trans*-acting regulator of DHS 4, and, critically, a manageable number of false positives. Utilizing a variety of curated databases and manual searches, the minimally-required 13 bp sequence (which was extended by 6 bp in both directions, see Materials and Methods) was scanned for binding sites and high-scoring position weight matrix (PWM) matches for hundreds of transcription factors (Fig. S3A). Of the 42 highly-scoring transcription factors, none were expressed consistently in OTX2-expressing medulloblastomas as detected by SAGE [35], with the sole exception of OTX2 itself. Notably, the minimally-required region of DHS 4 contains a highly-scoring OTX2 binding motif (p = 1.36e^−4^)(Fig. 3G) that exhibits a prominent DNase footprint [37] in medulloblastoma cells (Fig. S3B), strongly implicating direct binding of OTX2 protein to this region.

**Figure 4:**
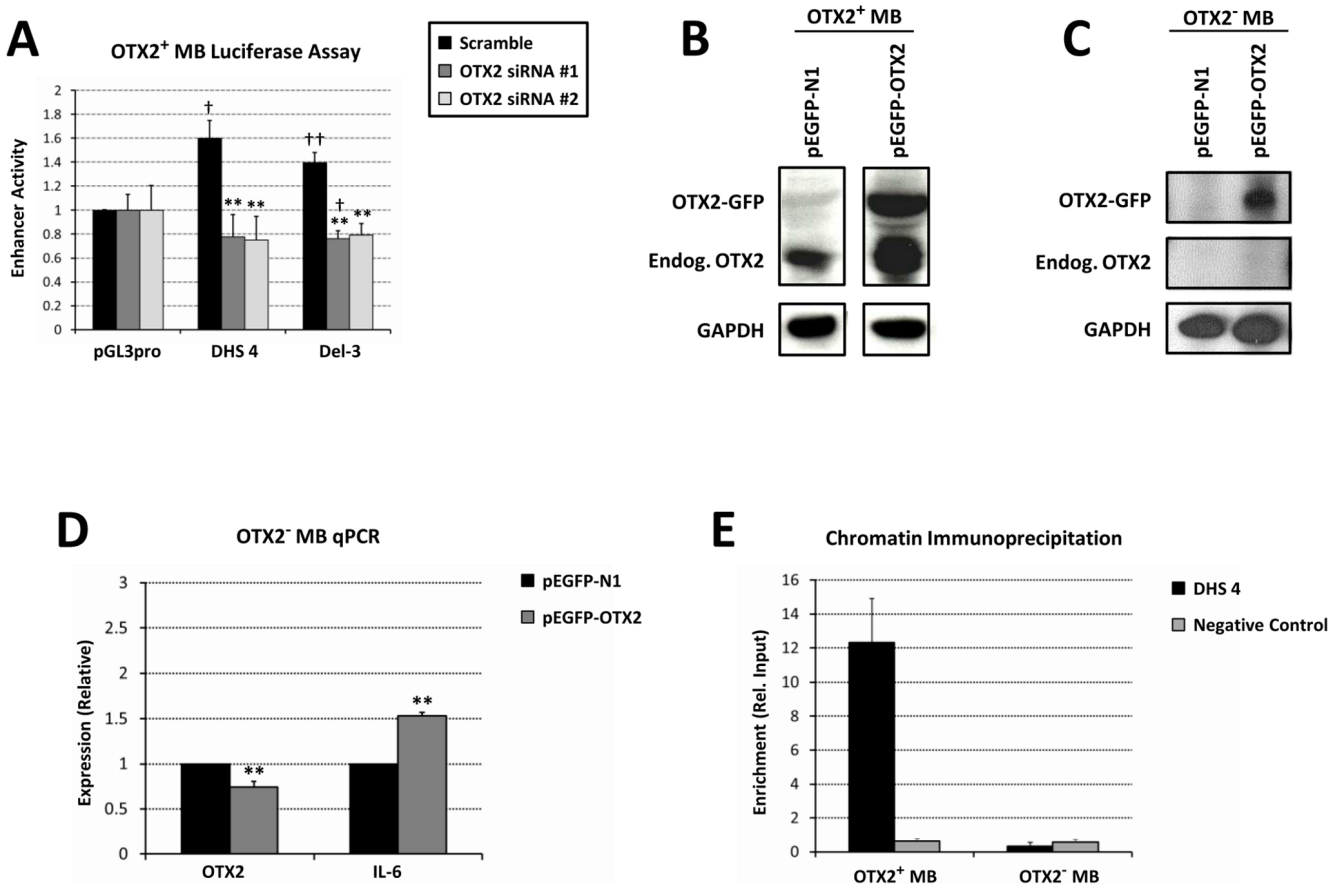
OTX2 regulates its own expression through the DHS 4 enhancer. (A) Luciferase assay for enhancer activity of DHS 4 in OTX2-expressing medulloblastoma cells treated with OTX2 siRNA. (B) Western blotting for ectopic and endogenous OTX2 following transfection of (B) OTX2-expressing or (C) OTX2-nonexpressing medulloblastoma cells with EGFP-tagged OTX2. (D) RT-qPCR demonstrating induction of the OTX2 target gene IL-6 in OTX2-nonexpressing medulloblastoma cells transfected with EGFP-tagged OTX2. (E) Chromatin immunoprecipitation of endogenous OTX2 in OTX2-expressing and -nonexpressing medulloblastoma cells. *p<0.05 relative to scramble siRNA (A) or vector control (D), **p<0.01 relative to scramble siRNA (A) or vector control (D), †p<0.05 relative to pGL3pro, ††p<0.01 relative to pGL3pro, Student's t-test. Error bars indicate standard deviation.

We then sought to determine the requirement of OTX2 for DHS 4 enhancer activity. Knockdown of OTX2 with distinct siRNA's completely abolished enhancer activity of DHS 4 in OTX2-expressing medulloblastoma cells (Fig. 4A). As a control for OTX2 knockdown, we utilized the OTX2-regulated fragment of the *MYC* promoter, Del-3 [5], cloned into the pBV reporter plasmid, which responded similarly to OTX2 inhibition.

To determine the ability of OTX2 to enhance its own expression from the native *OTX2* locus, we then overexpressed EGFP-tagged OTX2, which migrates more slowly during SDS-PAGE electrophoresis, to determine the effect of ectopic OTX2 upon expression of the endogenous *OTX2* gene. In OTX2-expressing medulloblastoma cells, we found that ectopic OTX2 indeed enhanced the expression of endogenous OTX2 (Fig. 4B). In contrast, ectopic OTX2 did not enhance endogenous OTX2 expression in medulloblastoma cells that do not normally express OTX2 (Fig. 4C), indicating that the appropriate cellular context is required for OTX2 autoregulation. Induction of the OTX2 target gene *IL-6* (Fig. 4D) [6], [7] in this experiment suggests that ectopic OTX2 is indeed functional in the transfected cells. As validation that autoregulation is not an overexpression artifact, chromatin immunoprecipitation of endogenous OTX2 specifically enriched the DHS 4 fragment in OTX2-expressing medulloblastoma cells (Fig. 4E).

### *OTX2* promoter methylation is sufficient to inhibit OTX2 expression in medulloblastoma

**Figure 5:**
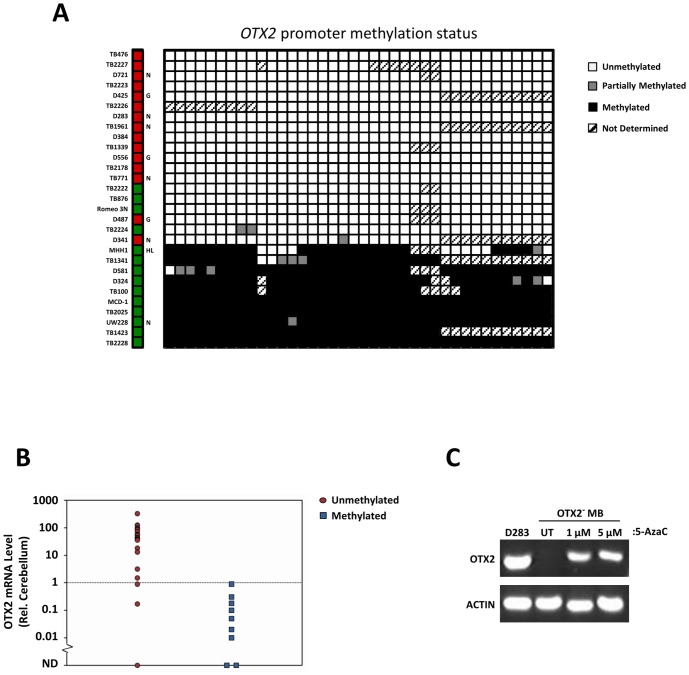
Methylation of the *OTX2* promoter is sufficient to suppress OTX2 in medulloblastoma. (A) Representative methylation patterns of medulloblastoma samples, with samples organized by row and each CpG location organized in columns. Leftmost column indicates OTX2 expression status (red, OTX2-expressing; green, OTX2-nonexpressing), letters to the right of this column indicate *OTX2* copy number status (N, normal; G, gained; HL, heterozygous loss), if known [5], [19], [32], [33]. (B) OTX2 mRNA level, as determined by RT-qPCR, in tumors exhibiting either a methylated or unmethylated *OTX2* promoter. ND, not detected. (C) Semi-quantitative RT-PCR of OTX2-nonexpressing cells treated with the indicated concentrations of 5′-aza-2′-deoxycytidine for 5 days. D283 medulloblastoma cells serve as a positive control for OTX2 detection.

Independent of distal chromatin accessibility and the activity of *trans*-acting factors, OTX2 expression could also be regulated by promoter DNA methylation. To this end, we determined the methylation status of *OTX2* by bisulfite sequencing in a cohort of medulloblastomas. The *OTX2* locus is tiled with a number of CpG islands, the largest of which spans the region 200 bp to 2.2 kb downstream of the OTX2 long isoform TSS (Fig. 1A). This CpG island is distinct from proximal DHS sites described herein. Bisulfite sequencing of this element revealed the binary nature of *OTX2* promoter methylation status (Fig. 5A, Table S2), with 30% of tumors exhibiting promoter methylation and 70% exhibiting a predominately unmethylated promoter (n = 33), which was supported by methylation-specific PCR (Table S2). There was a clear association between promoter methylation and *OTX2* repression (R = −0.5755, *p* = 0.0005, Spearman's rho, Fig. 5B), with frequent OTX2 expression (19/23; ≥2-fold increased relative to normal cerebellum by qPCR; see red boxes in Fig. 5A) among tumors harboring an unmethylated *OTX2* promoter and absent OTX2 overexpression (0/10; see green boxes in Fig. 5A) among samples exhibiting a methylated promoter (Fig. 5B). Finally, we determined that reversal of promoter methylation by treatment with 5-aza-2′deoxycytidine partially rescues OTX2 expression (Fig. 5C), indicating that promoter methylation actively represses *OTX2*. In sum, these results indicate that *OTX2* promoter methylation is sufficient for gene repression in medulloblastoma, and that an unmethylated promoter is necessary (but not sufficient) to permit OTX2 expression. Thus, OTX2 expression in medulloblastoma requires both positively-acting transcriptional regulators as well as a lack of *OTX2* promoter methylation.

### The chromatin landscape of *OTX2* in medulloblastoma resembles that of the embryonic brain

Given the accumulation of chromatin structure data for a variety of tissue contexts, we then sought to compare the regulatory landscape of *OTX2* in medulloblastoma with that of related CNS tissues. To this end, we mapped medulloblastoma DHS sites onto the mouse genome and investigated relevant chromatin characteristics of these elements in publically-available genome browser tracks [36], [38]. To determine chromatin states of DHS sites in the context of dynamic OTX2 regulation, we focused on embryonic day 14.5 (E14.5) whole brain (which includes forebrain, midbrain, and hindbrain) and adult cerebellum. This comparison revealed that regions orthologous to DHS sites 1, 4, 6, 7, and “R” exhibit DNase hypersensitivity in the E14.5 whole brain and, to a lesser extent, adult cerebellum (Fig. S4). As combinatorial histone modification patterns have been demonstrated to associate with distinct regulatory activities [39], predicted functions of regions orthologous to medulloblastoma DHS sites were determined (Table S1) using the parameters described in [39]. Specifically, the presence of H3K4me3 and H3K27Ac at open chromatin proximal (<2 kb) to a TSS is indicative of an active promoter, whereas H3K4me1 and H3K27Ac at open chromatin distal to a TSS is indicative of a strong enhancer. As expected, known enhancers driving forebrain and midbrain OTX2 expression exhibited strong H3K4me1 and H3K27Ac signals in the E14.5 brain (pink bars in Fig. S4), supporting the potential for chromatin state to predict regulatory function. Strikingly, chromatin states of mouse embryonic whole brain or adult cerebellum (Table S1, Fig. S4) accurately predicted the functional status of DHS sites in medulloblastoma (as assayed in Fig. 3C–D), with the one exception of DHS 5 (Table S1). These results indicate that a cell population in the developing brain, and to a lesser extent that of the mature cerebellum, exhibits an *OTX2* regulatory landscape similar to medulloblastoma.

### DHS “R” mediates retinoid-induced OTX2 repression in medulloblastoma

**Figure 6:**
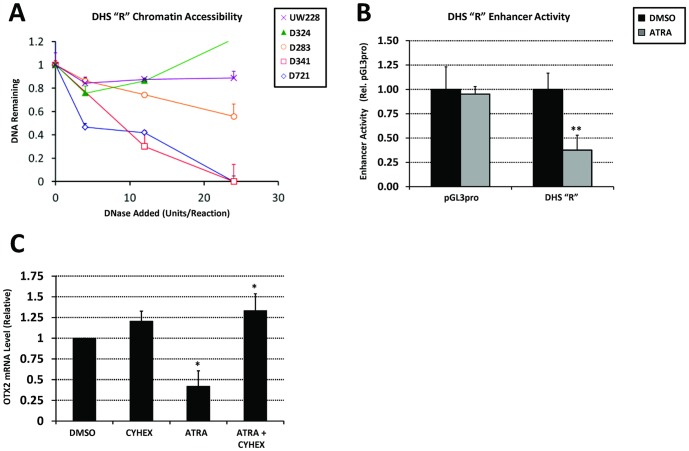
DHS “R” mediates retinoic acid-induced OTX2 repression. (A) DNase sensitivity of DHS “R” in three OTX2-expressing (D283, D341, D721) and two OTX2-nonexpressing (D324, UW228) medulloblastoma cell lines. (B) Luciferase assays measuring enhancer activity of DHS “R” following treatment with 2 µM all-*trans* retinoic acid (ATRA) for 24 hours. (C) OTX2 mRNA level as determined by qPCR of medulloblastoma cells treated with 2 µM all-*trans* retinoic acid (ATRA) and/or 35 µM cycloheximide (CYHEX) for 8 hours. *p<0.05 relative to DMSO, **p<0.01 relative to DMSO, Student's t-test. Error bars indicate standard deviation.

Retinoids are known to repress Otx2 during anterior-posterior embryonic patterning [18] and when added to cultured medulloblastoma cells [19], [20], and thus have been considered a potential therapeutic agent for medulloblastoma. Comparison of DHS sites among cell lines revealed a prominent DHS (DHS “R”) in retinoblastoma located between DHS sites 3 and 4 (Figs. 1 and S1A) that is present at variable intensity in medulloblastoma cells (Fig. 6A) and whose ortholog is bound in the mouse eye by the retinal transcription factor Crx (Fig. S4) [40]. Considering the potential role of retinoids in regulating OTX2 in the eye, these observations led us to investigate the role of the DHS “R” regulatory element in retinoid-induced repression of OTX2 in medulloblastoma. Luciferase assays measuring the transcriptional regulatory activity of DHS “R” revealed that this element represses transcription from a minimal promoter following retinoic acid treatment (Fig. 6B). These results are consistent with a model in which the repressor activity of DHS “R” is poised in medulloblastoma cells and becomes activated following retinoid treatment. Retinoid-induced OTX2 repression requires translation of an unknown intermediate (Fig. 6C) as indicated by circumvention of OTX2 repression in the presence of a translation inhibitor, cycloheximide (CYHEX), suggesting that OTX2 is not directly regulated by retinoic acid receptors but rather through induction of a currently-unknown transcriptional repressor. These observations form a model in which retinoids stimulate expression of a protein that mediates repressor activity of DHS “R”.

## Discussion

In this study, we have implemented a genomewide approach to describe for the first time the mechanisms of basal and retinoid-repressed *OTX2* transcription in medulloblastoma, thus contributing to the understanding of critical events in both medulloblastoma tumorigenesis and its response to differentiation therapy.

While *OTX2* genomic copy number gain is known to drive aberrant OTX2 expression in a subset (∼21%) of medulloblastomas [5], [19], [32], [33], alternative mechanisms of OTX2 overexpression have not been previously described. Here we have revealed various complementary observations that build a model of basal OTX2 overexpression in the absence *OTX2* genomic alterations. First, OTX2 expression associates with accessible chromatin regions and unmethylated promoter DNA at the *OTX2* locus, indicating that DNA accessibility limits OTX2 expression to a restricted set of normal and transformed tissues. Additionally, OTX2 protein directly activates the only *OTX2* regulatory element that is specifically active in OTX2-expressing medulloblastomas. Finally, *OTX2* enhancers in medulloblastoma are marked by activating histone modifications in the embryonic brain. These observations suggest that an OTX2 autoregulatory loop engages developmental enhancers to drive aberrant OTX2 expression in medulloblastoma.

Could OTX2 expression be inherited from the medulloblastoma cell of origin? OTX2 is expressed in the presumptive hindbrain [9], the tissue from which medulloblastoma arises, and here we have revealed parallels between regulatory element activities in medulloblastoma and corresponding histone modifications in the developing brain. An alternative cause of OTX2 expression in these tumors would be that OTX2 is upregulated over the course of transformation through the acquisition of developmental or novel regulatory regions, as recently described for breast cancer or T-cell leukemia [36]. However, given that OTX2 itself is required for activation of the key DHS 4 enhancer in medulloblastoma, it would be unlikely for OTX2 to be transcriptionally re-activated through this enhancer in the absence of OTX2 protein. Furthermore, we have demonstrated that medulloblastoma cells not expressing OTX2 are refractory to *OTX2* transcriptional activation through *trans*-acting factors. Thus, failure to repress OTX2 during development could be the critical determinant of OTX2 expression status in medulloblastoma. Notably, this prediction does not rule out the involvement of mediators that cooperate with OTX2 to modulate DHS 4 activity. The role of such factors, including chromatin modifiers and OTX2-interacting proteins, remains to be determined.

The identification of a positive feedback loop maintaining OTX2 expression in medulloblastoma suggests that disruption of this loop is a requisite step in the repression of *OTX2* during neural development. The mechanisms of developmental OTX2 repression that may fail during medulloblastoma tumorigenesis are currently unclear. The existence of medulloblastomas that lack both OTX2 expression and promoter methylation indicates that DNA methylation is unlikely the initiator of OTX2 repression during development, but rather serves as reinforcement of a repressive chromatin state, as previously demonstrated for other loci [24], [41]. Known developmental repressors of OTX2 in the hindbrain include Gbx2 [42] and Fgf8 [43]; however, these genes are rarely expressed in established medulloblastomas (Fig. S5) [35]. Although the dynamic interplay of these mediators of OTX2 repression during medulloblastoma initiation are currently unknown, an improved understanding of the cellular origins of this tumor and the developmental regulation of OTX2 therein should clarify these remaining questions regarding the molecular pathogenesis of OTX2-driven medulloblastoma.

The importance of Otx2 to myriad aspects of vertebrate patterning [9], [14], [44] has led to intense investigation of its regulation throughout development. These efforts have led to the mapping of various functional enhancers mediating Otx2 expression at many developmental stages (Summarized in Fig. S3 and Table S3) [11]–[13], [25], [26], [45]. In many cases, the *trans*-acting factors responsible for stage- and domain-specific Otx2 expression have also been identified, and for the midbrain/hindbrain boundary, a definitive model of Otx2 regulation has been constructed [26]. However, the expression patterns of known *trans*-acting Otx2 developmental regulators cannot in any case explain the expression pattern of OTX2 in medulloblastoma (Fig. S5), and mapping all previously-described *Otx2* developmental enhancers onto the human genome reveals minimal overlap between these enhancers and medulloblastoma DHS sites (Fig. S1A). Conversely, when functionally-validated medulloblastoma DHS sites were mapped onto the mouse genome, these sites frequently overlapped with signatures of promoter or enhancer elements in the embryonic brain and adult cerebellum (Fig. S4). It is likely that these elements are functional but have not yet been characterized with reporter assays at this domain and developmental stage. Thus, the transcriptional regulatory elements identified here have not been previously characterized in mouse development or otherwise.

The effect of retinoids upon medulloblastoma has been appreciated for some time [19]–[21], [46], and clinical trials implementing retinoid treatment as an adjuvant therapy for medulloblastoma are underway. However, the resistance of medulloblastoma cells to retinoids in some experimental settings [20] necessitates further study of the intermediates of retinoid-induced growth arrest in this tumor type. Such investigations could identify alternative or cooperative approaches to inducing the downstream antitumor effects of retinoids, such as BMP2 secretion and OTX2 repression [20], [21]. In this study, we have identified the transcriptional regulatory element responsible for OTX2 repression by retinoids. Although the *trans*-acting factor mediating the repressor activity of this element is unknown, Crx has been shown to bind to this regulatory element in the mouse eye (Fig. S4) [40]. Future studies implementing the approach described herein would clarify the role of CRX or other transcription factors in mediating the effect of retinoids upon OTX2.

The identification of *cis*-regulatory elements in the genome is of broad interest and has countless potential applications in basic and translational research. Through the ENCODE project, massive efforts have been undertaken to characterize the regulatory landscape of the human genome in various cell types and disease states [24], [36], [37], [47]. Considering the wealth of chromatin structure data generated through such endeavors, the extension of these studies into validated regulatory relationships has only just begun. Determining the underlying transcriptional regulation of single genes, certainly a frequently-pursued goal, requires a focused approach combining reporter assays, transcription factor motif searches, and functional validation, as demonstrated by our current studies. Unsurprisingly, there are few successful examples of *de novo* identification of distal-acting transcriptional regulators in mammalian cell lines, with many such approaches relying upon sequence conservation as a guide [48] or the concerted efforts undertaken to study some of the best-characterized loci in the genome, such as *CFTR* [49] and *IgH* [50]. Here we have implemented a hybrid approach combining genome-wide DNase-seq data with conventional gene regulatory assays to identify and functionally define long-range regulatory elements mediating transcription of a gene of interest. Our work thus serves as a guide for future studies intended to validate transcriptional regulatory relationships implicated through growing datasets from the ENCODE project. This approach will be further strengthened as transcription factor binding site motifs are accurately deciphered for all of the ∼1500 transcription factors encoded in the human genome. Additionally, the expansion of cell lines and tissue types assessed for DNase hypersensitivity or similar broadly-informative assays such as FAIRE-seq or Hi-C will enable such studies of essentially any gene under myriad physiological conditions.

## Supporting Information

Figure S1 **Comparison of** ***OTX2*** **transcriptional landscapes in various OTX2-expressing cell types.** (A) DNase hypersensitivity landscape in OTX2-expressing and -nonexpressing cell lines, wide viewing window. From top to bottom, tracks are as follows: known mouse enhancers (as summarized in Table S3), UCSC Genes, D341 medulloblastoma (Medullo) cells, D721 medulloblastoma cells, Weri-Rb retinoblastoma cells from [36], SK-N-MC Ewing's Sarcoma cells from [36], H1 embryonic stem (ES) cells, MultiZ vertebrate alignments, PhyloP vertebrate conservation. Green bars indicate regions of consistent and robust DNase hypersensitivity in the two medulloblastoma cell lines. The grey bar indicates DHS “R”, which is a DHS site in retinoblastoma cells and is utilized as a regulatory element in the retina. (B) CTCF binding in OTX2-expressing cell lines as determined by ChIP-seq. Grey bars indicate CTCF-bound elements, and the green bar indicates CTCF binding at medulloblastoma DHS 3. (C) Pol2 binding in cell lines predominately expressing short or long isoforms of OTX2 (ES cells and medulloblastoma, respectively) as determined by ChIP-seq.
(PDF)

Figure S2 **Validation of medulloblastoma DHS sites in a cohort of OTX2-expressing and -nonexpressing cells.** Refers to Fig. 2. Nuclei of medulloblastoma cell lines were treated with increasing concentrations of DNase, and then the relative proportion of DNA remaining at the indicated regions were determined by qPCR. Open and filled markers indicate OTX2-expressing and -nonexpressing cell lines, respectively. (A) DHS 1, (B) DHS 2, (C) DHS 3, (D) DHS 4, (E) DHS 5, (F) DHS 6, (G) DHS 7, and (H) positive control DHS. Rxn, Reaction. Error bars indicate standard deviation.
(PDF)

Figure S3 **Identification of transcription factor binding sites in DHS 4.** (A) Summary of database search results for transcription factor binding motifs in the critical 13 bp and 12 bp flanking sequence (see Materials and Methods) of DHS 4. (B) Evidence for a DNase footprint at the OTX2 binding motif of DHS 4 in medulloblastoma cells. “Density” tracks refer to smoothened data (as described in [23]), whereas “Raw” tracks refers to data without smoothening.
(PDF)

Figure S4 **Cross-species comparison of the** ***OTX2*** **transcriptional landscape in medulloblastoma and CNS tissues.** Chromatin structure of the mouse *Otx2* locus. From top to bottom, tracks are as follows: known *Otx2* developmental enhancers as determined by transgenic mouse reporter assays, regions of homology to medulloblastoma DHS sites, Refseq genes, CRX ChIP-seq of mouse eye [40], E14.5 whole mouse brain DNase-seq, Pol2 ChIP-seq, H3K4me1 ChIP-seq, H3K4me3 ChIP-seq, H3K27Ac ChIP-seq, CTCF ChIP-seq, from [38] and the UCSC Genome Browser, adult (8-week-old) cerebellum DNase-seq, Pol2 ChIP-seq, H3K4me1 ChIP-seq, H3K4me3 ChIP-seq, H3K27Ac ChIP-seq, CTCF ChIP-seq, from [38] and the UCSC Genome Browser, Multiz vertebrate alignment, PhyloP vertebrate conservation. Green bars indicate medulloblastoma DHS sites harboring marks of enhancer or promoter elements, pink bars indicate known brain enhancers, grey bar indicates DHS “R”.
(PDF)

Figure S5 **Assessment of gene expression data for** ***OTX2*** **regulators proposed in the literature.** (A) Characteristics of each proposed regulator, direction (activator or repressor) of regulation, the proposed tissue domain of *OTX2* regulation, *cis*-element regulated (of elements mapped in Fig. S4), transcription factor family, reference, *p*-value from two-tailed Student's t-test comparing expression in OTX2-positive (n = 19) and OTX2-negative (n = 8) medulloblastomas as determined by SAGE, Spearman's rho correlation R value and *p*-value (as indicated in parentheses) with OTX2 in medulloblastomas as determined by SAGE [35]. *previously implicated in medulloblastoma: Chd7 [3], Lef1 [2], Tcf7 [2], Smad3 [51], Hes1 [52], Suz12 [3]. References for developmental regulation of Otx2: Chd7 [53], Oct4/Pou5f1 [54], Rax [55], Lef1 [26], Yy1 [56], Brn1–4 [26], Tcf7 [13], Smad3 [57], Sox2 [54], Gbx2 [26], Eed [58], Crx [59], Hes1 [55], Suz12 [60], Snail [61]; †expression pattern reversed relative to expected relationship based on directional (activator or repressor) activity; ND, not determined; V/M/C, Anterior visceral endoderm, anterior mesendoderm, cephalic neural crest; FB/MID, forebrain/midbrain; MB, medulloblastoma. (B–D) Expression patterns of the most highly significant repressors and activators sorted by OTX2 expression level demonstrating that comparisons or correlations are largely driven by outliers in each case (as indicated with asterisks). MYC expression pattern is provided in (E) as an example of a gene exhibiting a known regulatory relationship [5] and correlation with OTX2 (R = 0.49, *p* = 0.009, Spearman's rho).
(PDF)

Table S1 **Summary of DHS characteristics.**
(PDF)

Table S2 **Bisulfite sequencing and methylation-specific PCR results for 33 medulloblastomas.**
(XLS)

Table S3 **Genomic coordinates of known** ***OTX2*** **developmental regulators.**
(XLSX)
