# Supplementary figures and images for: Chromatin Accessibility Mapping Identifies Mediators of Basal Transcription and Retinoid-Induced Repression of OTX2 in Medulloblastoma
Source: PLoS One. 2014 Sep 8;9(9):e107156. doi: 10.1371/journal.pone.0107156 (PMC4157845; doi:10.1371/journal.pone.0107156)

# Figure S1

**A**

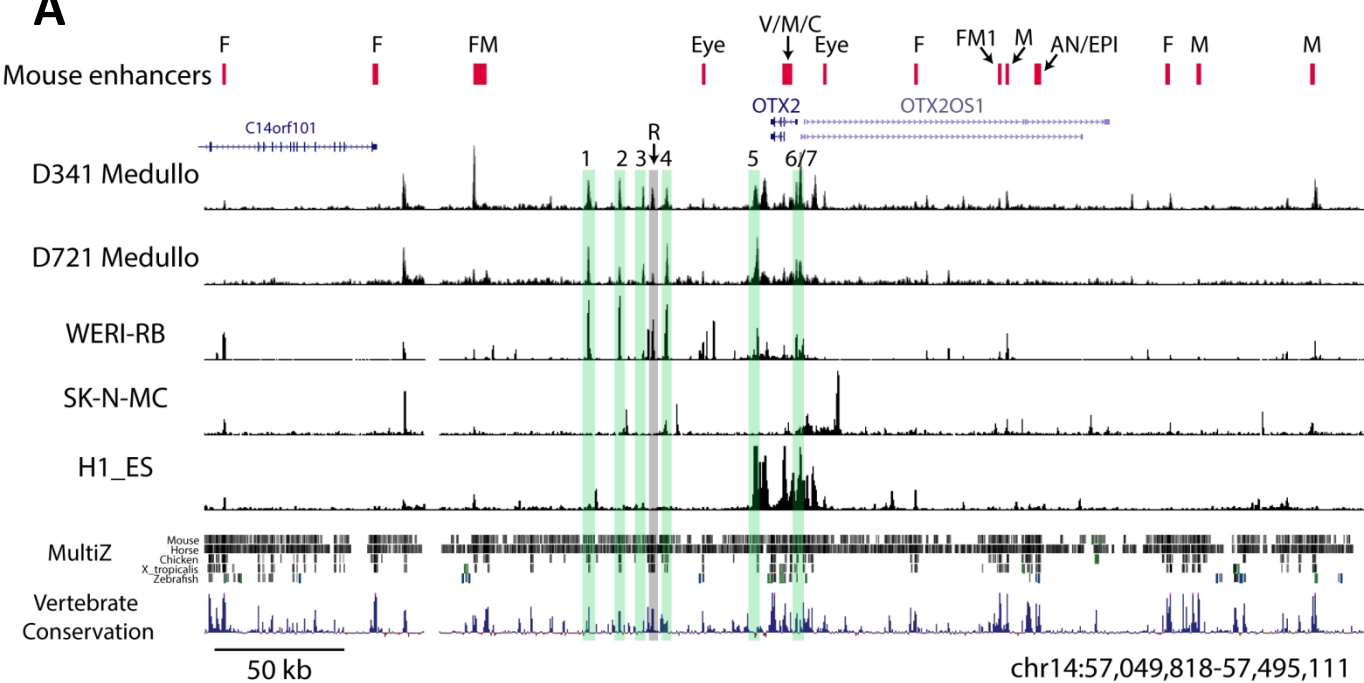

**B**

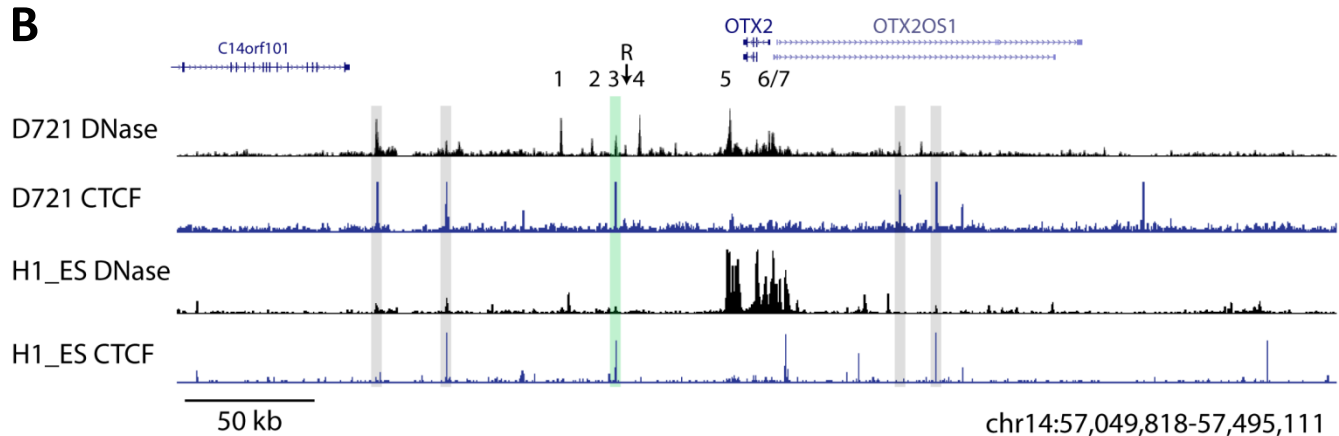

**C**

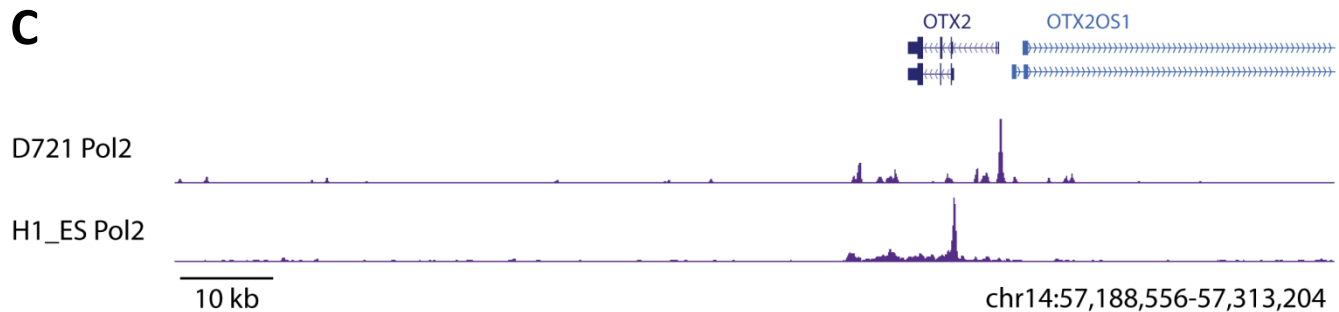

Supplement: Figure S1 — Comparison of OTX2 transcriptional landscapes in various OTX2-expressing cell types. (A) DNase hypersensitivity landscape in OTX2-expressing and -nonexpressing cell lines, wide viewing window. From top to bottom, tracks are as follows: known mouse enhancers (as summarized in Table S3), UCSC Genes, D341 medulloblastoma (Medullo) cells, D721 medulloblastoma cells, Weri-Rb retinoblastoma cells from [36], SK-N-MC Ewing's Sarcoma cells from [36], H1 embryonic stem (ES) cells, MultiZ vertebrate alignments, PhyloP vertebrate conservation. Green bars indicate regions of consistent and robust DNase hypersensitivity in the two medulloblastoma cell lines. The grey bar indicates DHS “R”, which is a DHS site in retinoblastoma cells and is utilized as a regulatory element in the retina. (B) CTCF binding in OTX2-expressing cell lines as determined by ChIP-seq. Grey bars indicate CTCF-bound elements, and the green bar indicates CTCF binding at medulloblastoma DHS 3. (C) Pol2 binding in cell lines predominately expressing short or long isoforms of OTX2 (ES cells and medulloblastoma, respectively) as determined by ChIP-seq. (PDF) [file pone.0107156.s001.pdf]

# Figure S2

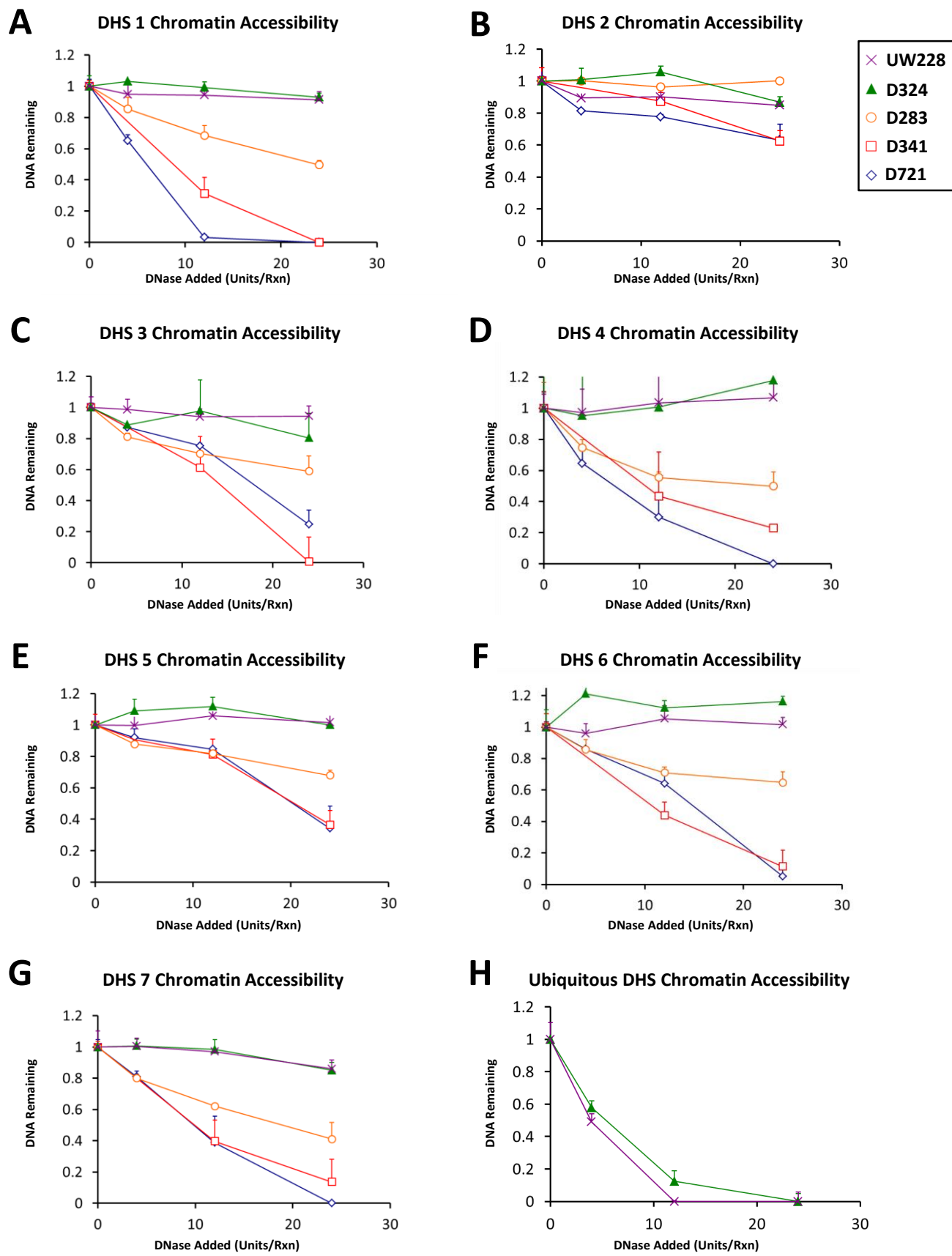

Supplement: Figure S2 — Validation of medulloblastoma DHS sites in a cohort of OTX2-expressing and -nonexpressing cells. Refers to Fig. 2. Nuclei of medulloblastoma cell lines were treated with increasing concentrations of DNase, and then the relative proportion of DNA remaining at the indicated regions were determined by qPCR. Open and filled markers indicate OTX2-expressing and -nonexpressing cell lines, respectively. (A) DHS 1, (B) DHS 2, (C) DHS 3, (D) DHS 4, (E) DHS 5, (F) DHS 6, (G) DHS 7, and (H) positive control DHS. Rxn, Reaction. Error bars indicate standard deviation. (PDF) [file pone.0107156.s002.pdf]

# Figure S4

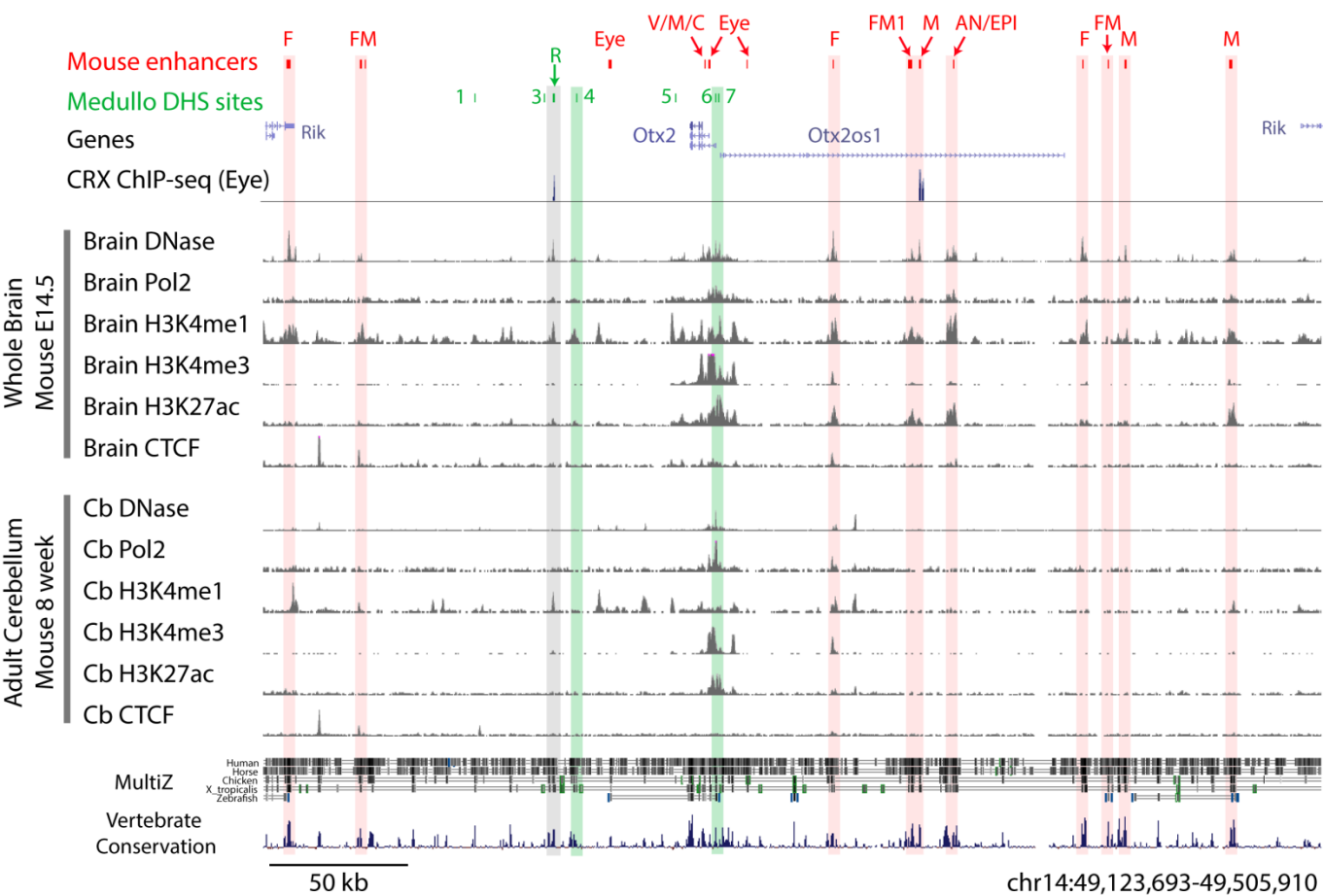

Supplement: Figure S4 — Cross-species comparison of the OTX2 transcriptional landscape in medulloblastoma and CNS tissues. Chromatin structure of the mouse Otx2 locus. From top to bottom, tracks are as follows: known Otx2 developmental enhancers as determined by transgenic mouse reporter assays, regions of homology to medulloblastoma DHS sites, Refseq genes, CRX ChIP-seq of mouse eye [40], E14.5 whole mouse brain DNase-seq, Pol2 ChIP-seq, H3K4me1 ChIP-seq, H3K4me3 ChIP-seq, H3K27Ac ChIP-seq, CTCF ChIP-seq, from [38] and the UCSC Genome Browser, adult (8-week-old) cerebellum DNase-seq, Pol2 ChIP-seq, H3K4me1 ChIP-seq, H3K4me3 ChIP-seq, H3K27Ac ChIP-seq, CTCF ChIP-seq, from [38] and the UCSC Genome Browser, Multiz vertebrate alignment, PhyloP vertebrate conservation. Green bars indicate medulloblastoma DHS sites harboring marks of enhancer or promoter elements, pink bars indicate known brain enhancers, grey bar indicates DHS “R”. (PDF) [file pone.0107156.s004.pdf]
