# Supplementary material for: Chromatin Accessibility Mapping Identifies Mediators of Basal Transcription and Retinoid-Induced Repression of OTX2 in Medulloblastoma
Source: PLoS One. 2014 Sep 8;9(9):e107156. doi: 10.1371/journal.pone.0107156 (PMC4157845; doi:10.1371/journal.pone.0107156)

Figure S3

A

Database	Rationale	# TF's represented	# Hits
JASPAR CORE	Curated database of vertebrate motifs	130	8
JASPAR HOMEO	Curated database of homeobox motifs	176	17
TRANSFAC TFblast	Curated database of vertebrate motifs	398	0
Consite	Integrates conservation scores	130	0
Manual alignment to motifs in Hu et al.	Confirmed *in vitro* interactions	201	8
Google Search	ID otherwise undocumented motifs	?	9

B

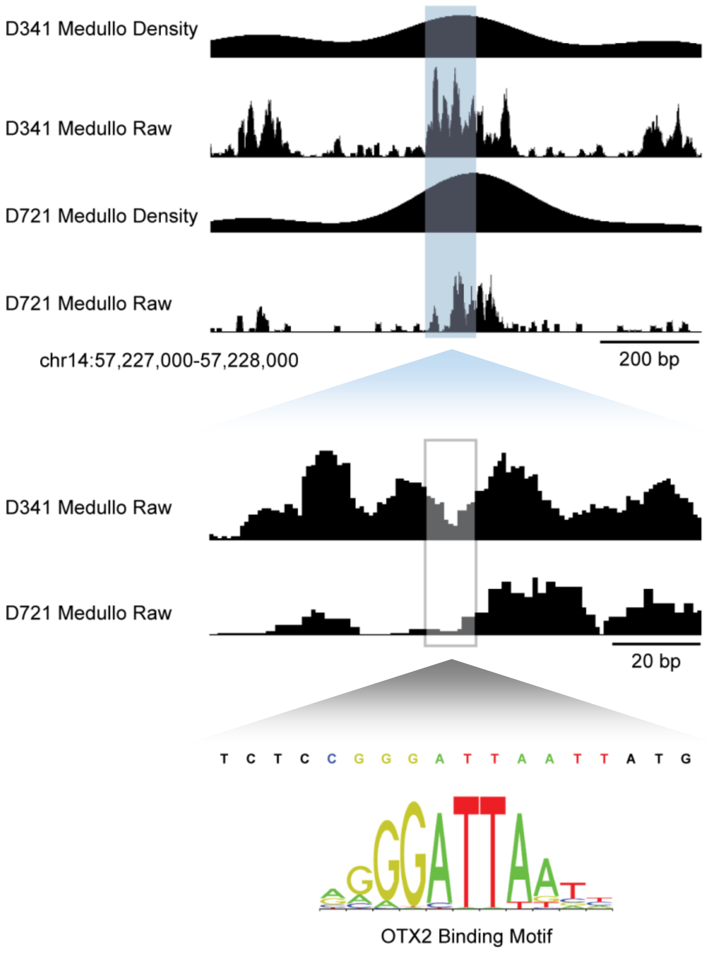

Supplement: Figure S3 — Identification of transcription factor binding sites in DHS 4. (A) Summary of database search results for transcription factor binding motifs in the critical 13 bp and 12 bp flanking sequence (see Materials and Methods) of DHS 4. (B) Evidence for a DNase footprint at the OTX2 binding motif of DHS 4 in medulloblastoma cells. “Density” tracks refer to smoothened data (as described in [23]), whereas “Raw” tracks refers to data without smoothening. (PDF) [file pone.0107156.s003.pdf]
