# Supplementary material for: Chromatin Accessibility Mapping Identifies Mediators of Basal Transcription and Retinoid-Induced Repression of OTX2 in Medulloblastoma
Source: PLoS One. 2014 Sep 8;9(9):e107156. doi: 10.1371/journal.pone.0107156 (PMC4157845; doi:10.1371/journal.pone.0107156)

Figure S5

A

Candidate Regulator	Direction	Domain	*cis*-element	Family	Reference	OTX2-positive v negative t-test	Spearman's rho (p-value)
Chd7*	Activator	Inner Ear	ND	ATP-dependent helicase	Hurd et al. 2010	0.203	0.30 (0.013)
Oct4/Pou5f1	Activator	ES cells	ND	POU Homeodomain	Sharov et al. 2008	0.524	0.19 (0.011)
Rax	Activator	Eye	EYE	Paired-type homeodomain	Muranishi et al. 2011	0.968	0.031 (0.051)
Lef1*	Activator	FB/MID	FM1	HMG domain (Wnt effector)	Kurokawa et al. 2004a	0.994	0.012 (0.14)
Yy1	Activator	AN	AN & V/M/C	GLI-Krüppel	Takasaki et al. 2007	0.643	0.24 (0.20)
Brn2	Activator	FB/MID	FM1	POU Homeodomain	Inoue et al. 2012	0.618†	-0.17 (0.49)†
Tcf7*	Activator	FB/MID	FM1	HMG domain (Wnt effector)	Kurokawa et al. 2004a	0.103†	-0.34 (0.43)†
Sox2	Activator	ES cells	ND	Sry Box HMG	Sharov et al. 2008	< 0.001†	-0.25 (0.27)†
Smad3*	Activator	AN	ND	Receptor-regulated Smad	Jia et al. 2009	0.0495†	-0.21 (.29)†
Brn1	Activator	FB/MID	FM1	POU Homeodomain	Inoue et al. 2012	NA	0 (0.0088)
Brn4	Activator	FB/MID	FM1	POU Homeodomain	Inoue et al. 2012	NA	0 (0.0088)
Gbx2	Repressor	Hindbrain	FM1	Homeodomain	Inoue et al. 2012	0.125	-0.24 (0.051)
Eed*	Repressor	Posterior Embryo	ND	Polycomb Group	Schumacher et al. 1996	0.0640	-0.14 (0.56)
Crx	Repressor	Eye	DHS "R"	OTX-like homeobox	Hsiau et al. 2007, Corbo et al. 2010	0.161†	0.24 (0.19)†
Hes1*	Repressor	Eye	EYE	Hes family (Notch effector)	Muranishi et al. 2011	0.534	0.0026 (0.26)†
Suz12*	Repressor	ES Cells	ND	Polycomb Group	Pasini et al. 2007	0.151†	0.32 (0.039)†
Snail	Repressor	Posterior Embryo	ND	Zinc Finger	Carver et al. 2001	NA	0 (0.0088)

B

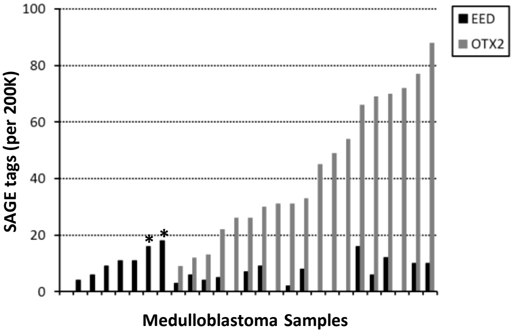

C

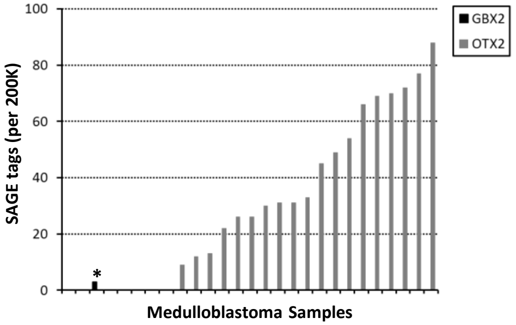

D

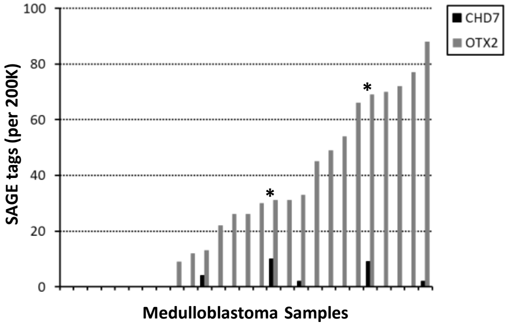

E

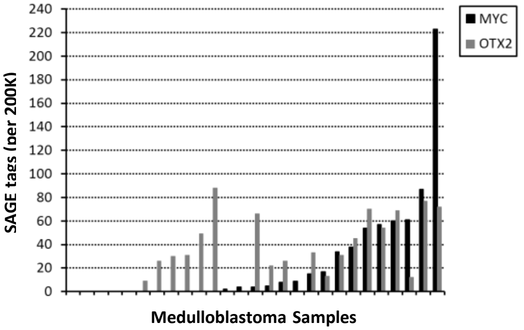

Supplement: Figure S5 — Assessment of gene expression data for OTX2 regulators proposed in the literature. (A) Characteristics of each proposed regulator, direction (activator or repressor) of regulation, the proposed tissue domain of OTX2 regulation, cis-element regulated (of elements mapped in Fig. S4), transcription factor family, reference, p-value from two-tailed Student's t-test comparing expression in OTX2-positive (n = 19) and OTX2-negative (n = 8) medulloblastomas as determined by SAGE, Spearman's rho correlation R value and p-value (as indicated in parentheses) with OTX2 in medulloblastomas as determined by SAGE [35]. *previously implicated in medulloblastoma: Chd7 [3], Lef1 [2], Tcf7 [2], Smad3 [51], Hes1 [52], Suz12 [3]. References for developmental regulation of Otx2: Chd7 [53], Oct4/Pou5f1 [54], Rax [55], Lef1 [26], Yy1 [56], Brn1–4 [26], Tcf7 [13], Smad3 [57], Sox2 [54], Gbx2 [26], Eed [58], Crx [59], Hes1 [55], Suz12 [60], Snail [61]; †expression pattern reversed relative to expected relationship based on directional (activator or repressor) activity; ND, not determined; V/M/C, Anterior visceral endoderm, anterior mesendoderm, cephalic neural crest; FB/MID, forebrain/midbrain; MB, medulloblastoma. (B–D) Expression patterns of the most highly significant repressors and activators sorted by OTX2 expression level demonstrating that comparisons or correlations are largely driven by outliers in each case (as indicated with asterisks). MYC expression pattern is provided in (E) as an example of a gene exhibiting a known regulatory relationship [5] and correlation with OTX2 (R = 0.49, p = 0.009, Spearman's rho). (PDF) [file pone.0107156.s005.pdf]
