# Supplementary material for: Chromatin Accessibility Mapping Identifies Mediators of Basal Transcription and Retinoid-Induced Repression of OTX2 in Medulloblastoma
Source: PLoS One. 2014 Sep 8;9(9):e107156. doi: 10.1371/journal.pone.0107156 (PMC4157845; doi:10.1371/journal.pone.0107156)
Supplement: Table S1 — Summary of DHS characteristics. (PDF) [file pone.0107156.s006.pdf]

Table S1

	DHS 1	DHS 2	DHS 3	DHS "R"	DHS 4	DHS 5	DHS 6	DHS 7
**Coordinates (hg19, chr14)**	57,197,081-57,197,624	57,208,951-57,209,448	57,218,296-57,218,708	57,221,649-57,222,405	57,227,265-57,227,738	57,261,971-57,262,522	57,276,973-57,277,519	57,278,349-57,278,688
**Position Rel. TSS (long isoform)**	+80,000	+68,000	+59,000	+55,000	+50,000	+15,000	0	-1000
**Differential Accessibility?**	Yes	No	Yes	Yes	Yes	Yes	Yes	Yes
**Predicted Function***	Enhancer	N.D.	Insulator	Strong Enhancer	Enhancer	Enhancer	Active Promoter	Active Promoter
**Actual Function**	Enhancer	N.D.	Insulator	Inducible Repressor	Enhancer	None Found	Promoter	Promoter/Enhancer
**Functional Specificity**	No	N.D.	N.D.	N.D.	Yes	N.D.	No	No
**Known Status in Human CNS**	RB DHS	RB DHS	No hypersensitivity	RB DHS	RB DHS	RB DHS (weak)	RB DHS	No hypersensitivity
**Known Status in Mouse CNS**	WB H3K4me1 (weak)	N.D.	No histone marks or DHS's	WB & CB H3K4me1, WB H3K27Ac	WB H3K4me1, WB H3K27Ac (weak)	WB & CB H3K4me1, WB H3K27Ac	WB & CB H3K4me3, WB & CB H3K27Ac	WB H3K4me1, WB & CB H3K27Ac
**Known TF Binding**			CTCF (MB, RB, ES)	CRX (eye)				
**Conservation (Alignment)**	Xenopus (88%, 98bp)	Xenopus (90%, 428bp)	Mammals (86%, 256bp)	Xenopus (86%, 52bp)	Xenopus (100%, 42bp)	Xenopus (80%, 159bp)	Zebrafish (75%, 35bp)	Mammals (95%, 335bp)
**Homology to Ms (% Identity)**	94%	94%	86%	90%	92%	90%	90%	95%
**Homology to Ms (Location, chr14)**	49,200,069-49,200,450	49,212,826-49,213,316	49,225,083-49,225,338	49,228,290-49,228,866	49,236,689-49,236,966	49,272,515-49,272,725	49,287,095-49,287,474	49,288,235-49,288,569
**Activity Detected in Mouse$**	None	None	None	Nasal Cavity**	Nasal Cavity**	Nasal Placode	N.D.	N.D.

"Known Status in Mouse CNS" refers to E14.5 forebrain or adult cerebellum from Ref. 38.
"Conservation (Alignment)" identity comparisons with mammals use the mouse genome as a reference sequence
***Based on classification system proposed in Ref. 39, predicted from histone status (H3K4me1, H3K4me3, H3K27Ac), DNase sensitivity, and CTCF binding in mouse embryonic whole brain or adult cerebellum from Ref. 38.**
$Assessed (at embryonic timepoints only) in Refs. 11-13.
****DHS 4 and DHS "R" reside in the in same fragment assayed**
Abbreviations: CNS, central nervous system; Ms, mouse; WB, whole embryonic brain; CB, cerebellum; RB, retinoblastoma; MB, medulloblastoma
